# Digital Therapy for Male LUTS: Results After Mid- and Longterm Follow-Up

**DOI:** 10.3390/jcm15062128

**Published:** 2026-03-11

**Authors:** Erik Krieger, Christian Gratzke, Kurt Miller, C. Patrick Papp, Laura Wiemer, Sandra Schönburg

**Affiliations:** 1 Charité, Department of Urology, University of Berlin, 10117 Berlin, Germany; 2 Department of Clinical Research, Kranus Health, 80331 Munich, Germany; 3 Department of Urology, University of Freiburg, 79106 Freiburg im Breisgau, Germany; 4 BG Klinikum Bergmannstrost, 06112 Halle (Saale), Germany; 5 Department of Urology and Kidney Transplantation, Martin Luther University, 06120 Halle (Saale), Germany

**Keywords:** digital therapy, male LUTS, BPH, OAB, long-term results

## Abstract

**Background**: The BEST study investigated the effectiveness of a 12-week digital treatment program for male LUTS. Here, we report on the long-term outcomes of the patients involved in this trial. **Methods**: The randomized controlled BEST trial enrolled 237 patients (intervention group, IG: n = 112, hereafter referred to as the direct intervention group [DIG]; control group, CG: n = 125, hereafter referred to as the postponed intervention group [PIG]). The intervention consisted of pelvic floor muscle training, behavioral training, completion of a micturition diary, bladder training, urge suppression techniques, fluid and dietary management, and structured educational content. Patients in the DIG received the intervention immediately, Patients in the PIG after a 12-week waiting period. Patients in both groups were offered the option to complete additional treatment cycles at their discretion. The primary endpoint was change from baseline in the International Prostate Symptom Score (IPSS). Secondary endpoints included the symptom severity (OAB-q SF1) and quality-of-life (OAB-q SF2) subscales of the Overactive Bladder Questionnaire, among others. Long-term follow-up assessments at 24, 36, and 48 weeks for participants in both study arms were prospectively specified in the study protocol. **Results**: Baseline data from 236 patients were available for the follow-up analyses. In a linear mixed-effects model, the fixed effect of time on IPSS was found to be statistically significant (F(4, 515.245) = 89.77, *p* < 0.001), indicating differences across measurement time points. Compared with the baseline, IPSS scores were lower at all subsequent follow-up assessments. The mean difference between the baseline and 12 weeks after was −6.32 points (95% CI: −7.60 to −5.04; *p* < 0.001). Differences between the baseline and 24 weeks (−7.81 points; 95% CI: −9.37 to −6.25; *p* < 0.001), baseline and 36 weeks (−8.62 points; 95% CI: −10.46 to −6.79; *p* < 0.001), and baseline and 48 weeks (−9.56 points; 95% CI: −12.66 to −6.46; *p* < 0.001) were also statistically significant. Comparable patterns of improvement were observed for both subscales of the OAB-q Short Form questionnaire. In a separate linear mixed-effects model, the fixed effect of time on IPSS after the discontinuation of app usage was not statistically significant (F(2, 19.750) = 0.01, *p* = 0.992), suggesting stable effects after discontinuation. **Conclusions**: Long-term outcomes of the structured app-based therapeutic program demonstrated that a multimodal digital intervention for male LUTS induces a rapid and clinically meaningful symptom reduction within the first 12 weeks, with consolidating and sustaining benefits over up to 48 weeks.

## 1. Background

Certified digital health applications in Germany are intended to detect or alleviate disease, support diagnostic processes, or accompany therapeutic interventions [1]. Demonstration of patient benefit in a randomized controlled trial is a prerequisite for reimbursement by statutory health insurance. Within this regulatory framework, the Bladder Emptying DiSorder Therapy (BEST) study demonstrated statistically significant and clinically meaningful improvements in male lower urinary tract symptoms, as assessed by the IPSS as well as the symptom (OAB-q SF1) and quality-of-life (OAB-q SF2) subscales of the Overactive Bladder Questionnaire [2]. These findings were further supported by predefined subgroup analyses [3] and post hoc evaluations of IPSS subdomains [4]. As the digital intervention was designed as a 12-week program, previously published analyses were limited to short-term outcomes. The present study provides a report of the prospectively planned follow-up at 24, 36, and 48 weeks.

## 2. Material and Methods

The BEST study was a two-arm, randomized controlled trial designed to evaluate the effectiveness of an app-based therapeutic intervention for improving lower urinary tract symptoms in men with benign prostatic hyperplasia and/or overactive bladder. Detailed inclusion and exclusion criteria have been reported elsewhere [2]. The present investigation reports on prospectively planned follow-up assessments. Between follow-up examinations, patients completed up to four treatment cycles. The original study period comprised 12 weeks, corresponding to one treatment cycle. Participants in the direct intervention group (DIG) received standard of care in addition to access to the digital therapy *Kranus Lutera*, comprising pelvic floor muscle training, behavioral training, completion of a micturition diary, bladder training, urge suppression techniques, fluid and dietary management, and structured educational content. Participants in the postponed intervention group (PIG) received standard of care alone and were granted access to the digital therapy after completion of the initial 12-week primary data collection period. All study participants were offered the option to undertake additional treatment cycles at their own discretion.

The analyses presented here were conducted on data provided by completers of the different follow-up examinations. Because examinations corresponding to different therapy phases were time-shifted for participants in the DIG and PIG, outcome data corresponding to respective therapy cycles were pooled. Selected analyses were stratified according to group allocation.

**Figure 1:**
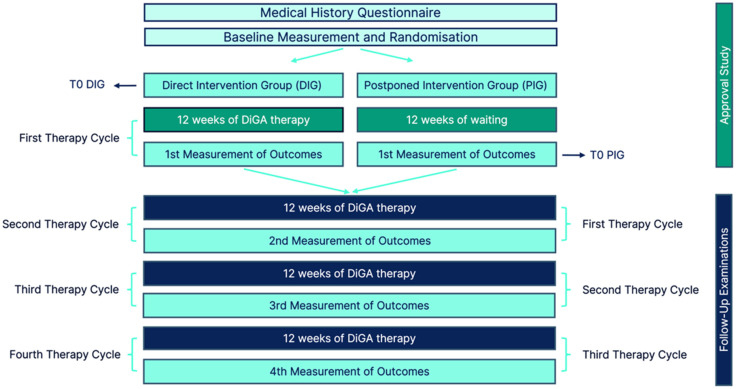
Schematic representation of the systematics of follow-up examinations.

Participants in both DIG and PIG were invited to complete follow-up questionnaires at 24, 36, and 48 weeks. Follow-up invitations were sent exclusively via email, containing a direct link to the online questionnaires. No additional methods to increase follow-up adherence were implemented. In the DIG, these assessments corresponded to outcomes after the second, third, and fourth therapy cycles, respectively. In contrast, the same time points reflected outcomes after the first, second, and third therapy cycles in the PIG (Figure 1). For both groups, the last assessment prior to initiation of the digital therapy was defined as the baseline (T0), corresponding to week 0 in DIG and week 12 in PIG. Subsequent time points T1, T2, T3, and T4 represent assessments conducted 12, 24, 36, and 48 weeks after initiation of the first therapy cycle. Data at T4 were available exclusively from participants in the DIG.

In this analysis, we examined the longitudinal course of LUTS severity using the validated IPSS questionnaire [5] and the OAB-q SF Part 1 symptom severity score, which was transformed to a 0–100 scale. In addition, health-related quality of life was assessed using the OAB-q SF Part 2 score (health-related quality of life), likewise transformed to a 0–100 scale [6]. For the IPSS and OAB-q SF Part 1, lower scores indicate lower symptom burden. In contrast, higher scores on OAB-q SF Part 2 reflect better health-related quality of life.

The study was reviewed and positively evaluated by the Ethics Committee of the Medical Faculty of Martin Luther University Halle-Wittenberg (Ethics Committee Approval No.: 2022-139) and the participating Ethics Committee of Albert Ludwig University Freiburg (Application No. EK-Freiburg: 23-1219-S1-AV) and registered in the German Clinical Trials Register (DRKS: DRKS00030935).

### Statistical Analyses

All analyses were conducted in an exploratory manner. Accordingly, only observed data were included in the statistical analyses, and no imputation procedures were applied to account for missing values. Given the substantial proportion of missing observations at later follow-up time points and the heterogeneous patterns of data availability across treatment cycles, imputation was considered inappropriate, as it would have relied on strong and unverifiable assumptions. The analytic strategy therefore focused on available measurements only, reflecting real-world usage and follow-up patterns. Consequently, results should be interpreted as descriptive and hypothesis-generating rather than confirmatory.

To evaluate longitudinal changes in symptom severity and health-related quality of life, the trajectories of the IPSS, the OAB-q SF1, and the corresponding quality-of-life subscale OAB-q SF2 were analyzed using linear mixed-effects models.

Each outcome score was specified as a dependent variable in separate models. Time since initiation of digital therapy was included as a fixed effect and modeled as a categorical variable with five levels: baseline, and 12, 24, 36, and 48 weeks after treatment start. To account for the correlation of repeated measurements within individuals and inter-individual variability in baseline symptom burden, patient-specific random intercepts were included using patient identification as the random factor.

To further characterize potential determinants of IPSS symptom trajectories, two additional mixed-effects models were specified. First, group allocation (DIG vs. PIG) and its interaction with time were included as fixed effects to evaluate whether overall symptom levels or temporal patterns differed between groups beyond the primary time effect. Second, adherence category and its interaction with time were incorporated as additional fixed factors to explore whether differences in app usage were associated with variation in IPSS outcomes over time. In both models, patient-specific random intercepts were retained to account for within-subject correlation of repeated measurements.

An additional analysis was performed to assess the persistence of treatment effects in IPSS scores after discontinuation of app-based therapy. Discontinuation was defined as non-usage of the app as a medium. Time since discontinuation of the digital intervention was included as a categorical fixed effect with three levels, corresponding to 12, 24, and 36 weeks after cessation of app use. Patient identifier was specified as a random effect to account for repeated measurements within individuals.

The use of linear mixed-effects modeling was chosen to appropriately handle the longitudinal data structure with varying numbers of completed treatment cycles and missing observations at follow-up time points. The analysis allowed all available measurements to contribute to the estimation of time-specific effects, even for participants with incomplete follow-up, thereby maximizing the use of the data without imputing missing values.

Model estimates are reported with the corresponding 95% confidence intervals, and statistical significance was assessed using two-sided tests with an alpha level of 0.05.

Patient characteristics, therapy adherence, and responder outcomes were analyzed descriptively. Responder analyses were based on two different definitions of minimal important difference (MID). One MID, derived from the work of Barry et al. [7], was defined using a cutoff value of 3 points on the IPSS. A second MID of 5.26 IPSS [8] points applied in the present analyses was calculated based on results from the BEST study.

All statistical analyses were performed with IBM SPSS Statistics software (version 31.0.0.0).

## 3. Results

### 3.1. Descriptive Results

#### 3.1.1. Patient Demographics

**Figure 2:**
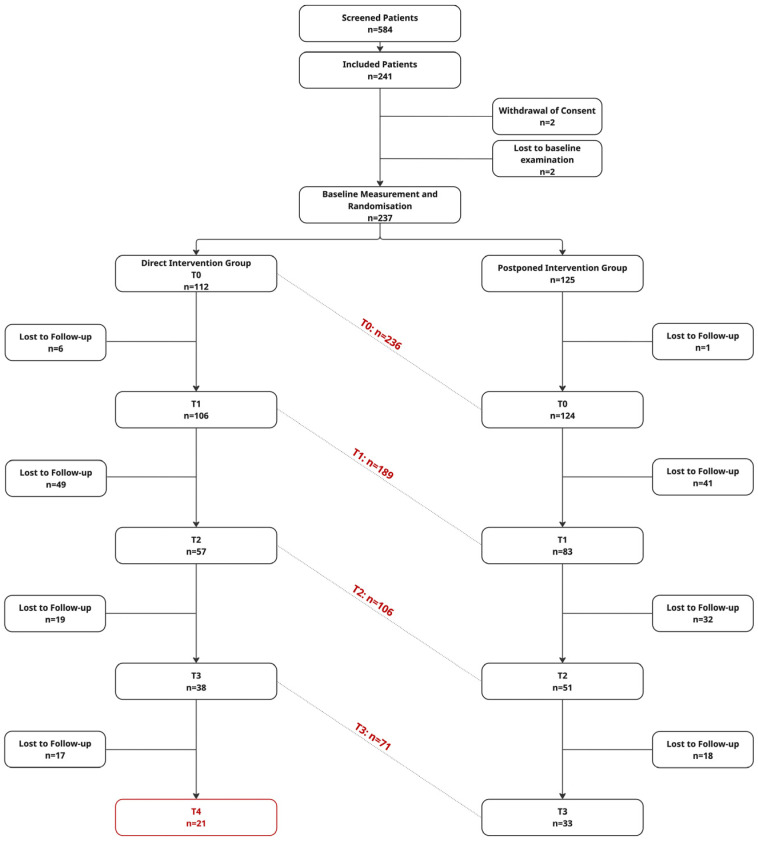
Number of patients contributing outcome data at each analysis time point, stratified by group allocation.

In total, data were available for 236 patients at T0 (one drop out in the PIG after their waiting period). Data from a total of 189 patients were available at T1. In addition to six dropouts from the DIG, there were 41 patients in the PIG who were lost to follow-up after their first therapy cycle. Of all the enrolled study participants, 79.75% (n = 189) completed the first post-intervention assessment. A total of 108 patients (45.57%) completed the second follow-up assessment, 71 patients (29.96%) the third, and 21 patients (8.86%) the fourth post-intervention assessment (Figure 2).

The study population consisted mainly of middle-aged and older men, with a mean age of 58.4 (±12.3) years. Most participants were 65 years of age or younger (n = 166, 70%), while 30% (n = 71) were older than 65 years.

With respect to clinical characteristics, prostatic hyperplasia represented the most frequently reported diagnosis, present in 46.4% (n = 110) of patients, followed by other specified disorders of the urinary bladder in 31.2% (n = 74). A combination of prostatic hyperplasia and other urinary bladder conditions was observed in 22.4% (n = 53) of the cohort. At the baseline, 29.1% (n = 69) of patients were currently receiving pharmacological treatment for bladder voiding dysfunction, and 18.8% (n = 45) reported prior pharmacological interventions for this condition, whereas previous prostate surgery was uncommon (n = 9, 3.8%).

Lifestyle characteristics included current smoking in 12.2% (n = 29) of patients, alcohol consumption in 62.0% (n = 147), and caffeine intake in 89.0% (n = 211). Comorbid conditions were reported by 57.0% (n = 135) of participants, most commonly arterial hypertension (n = 78, 32.9%), followed by erectile dysfunction (n = 34, 14.3%) and diabetes mellitus (n = 20, 8.4%). Other comorbidities, such as depression, heart failure, mental disorders, and renal insufficiency, occurred less frequently.

More detailed baseline characteristics have been reported elsewhere [2].

Baseline demographic and clinical characteristics were broadly comparable across the different measurement time points (Table 1). Mean age was similar across assessments, ranging from 58.4 (±12.3) years at baseline to 60.6 (±8.6) years at T3, with no consistent trend over time. The proportion of patients aged 65 years or younger in categories of age remained stable from baseline to T3, with patients aged 65 years or younger accounting for approximately two thirds of the cohort, while the proportion of patients older than 65 years ranged between 30% and 32.4%. At T4, the proportion of patients older than 65 decreased to 19%.

Mean body mass index (BMI) was comparable across all assessments and remained within a narrow range between 26.4 (±3.9) and 27.0 (±2.8) kg/m^2^.

Regarding disease characteristics, prostatic hyperplasia was the most frequently reported diagnosis at all time points, with proportions ranging from 46.4% at baseline to 57.1% at T4. The proportion of patients with other specified diseases of the urinary bladder decreased over time, from 31.2% at the baseline to 14.3% at T4. Patients with a combination of prostatic hyperplasia and other specified urinary bladder diseases accounted for approximately one fifth to one quarter of the cohort at all assessments.

The proportion of patients receiving medication for bladder voiding dysfunction was similar across the baseline, T1, T2, and T3 (approximately 29–33%), with a lower proportion observed at T4 (19.0%). Previous therapies for bladder voiding dysfunction were reported by 21.1% of patients at the baseline, with slightly higher proportions at later follow-up assessments, ranging from 22.2% at T1 to 28.6% at T4.

Mean IPSS values at baseline assessments showed a gradual increase from 17.57 (±6.3) at the baseline to 19.14 (±6.3) at T4.

#### 3.1.2. Adherence

**Figure 3:**
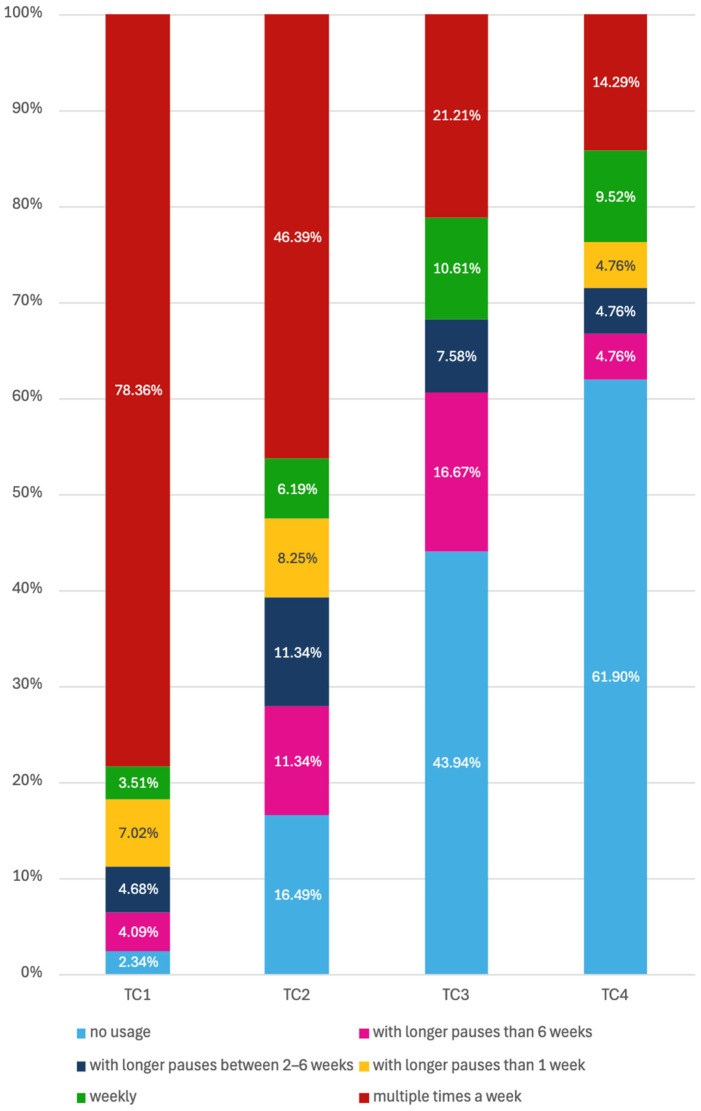
Distribution of app usage frequency categories across successive therapy cycles.

Patient engagement with the app differed across therapy cycles and showed a distinct time-dependent pattern characterized by decreasing use (Figure 3). During the first therapy cycle, most patients accessed the app multiple times per week (78.36%), whereas weekly use and longer intervals between sessions were infrequent. In the second cycle, the proportion of patients with multiple weekly accesses declined to 46.39%, accompanied by an increase in irregular use and non-use (16.49%). A further reduction in regular engagement was observed in the third cycle, with non-use reported by 43.94% of patients and multiple weekly access maintained by 21.21%. In the fourth cycle, app use was predominantly low, with 61.90% of patients reporting no usage and only 14.29% continuing to use the app multiple times per week. Taken together, app usage intensity decreased progressively across successive therapy cycles.

In the comparison between the DIG and the PIG, differences in app usage patterns across therapy cycles were observed (Table 2). During the first therapy cycle, adherence was high in both groups, with most patients accessing the app multiple times per week. This proportion was higher in DIG than in PIG (83.96% vs. 69.23%). No non-use was reported in DIG at this stage, whereas a small proportion of patients in PIG reported no app usage (6.15%). Irregular usage, defined as pauses longer than one week, was more frequently observed in PIG than in DIG.

In the second therapy cycle, app usage intensity decreased in both groups. The proportion of patients with multiple weekly accesses declined to 49.12% in DIG and 42.50% in PIG. Concurrently, the proportion of patients reporting no app usage increased in both groups, reaching 19.30% in DIG and 12.50% in PIG. Longer pauses between sessions, particularly interruptions of more than six weeks, were more frequently reported in PIG than in DIG.

During the third therapy cycle, non-use became the most common usage category in both groups, affecting a comparable proportion of patients (44.74% in DIG and 42.86% in PIG). While regular weekly usage was uncommon in both groups, access multiple times per week remained more frequent in DIG than in PIG (23.68% vs. 17.86%). No patients in either group reported app usage with pauses longer than one week but shorter than six weeks during this cycle.

By the fourth therapy cycle, overall app engagement was low. In DIG, 61.90% of patients reported no app usage, while smaller proportions reported longer pauses or intermittent use. A minority of DIG patients continued to access the app multiple times per week (14.29%). No corresponding PIG data were available for T4.

Overall, Table 2 illustrates a progressive shift from frequent app use toward irregular use and non-use across successive therapy cycles in both groups. Across all comparable cycles, frequent app access was consistently more common in DIG than in PIG, whereas longer pauses and non-use were observed at similar or higher proportions in PIG, particularly during the earlier therapy cycles.

### 3.2. Efficacy

#### 3.2.1. Primary Endpoint IPSS

**Figure 4:**
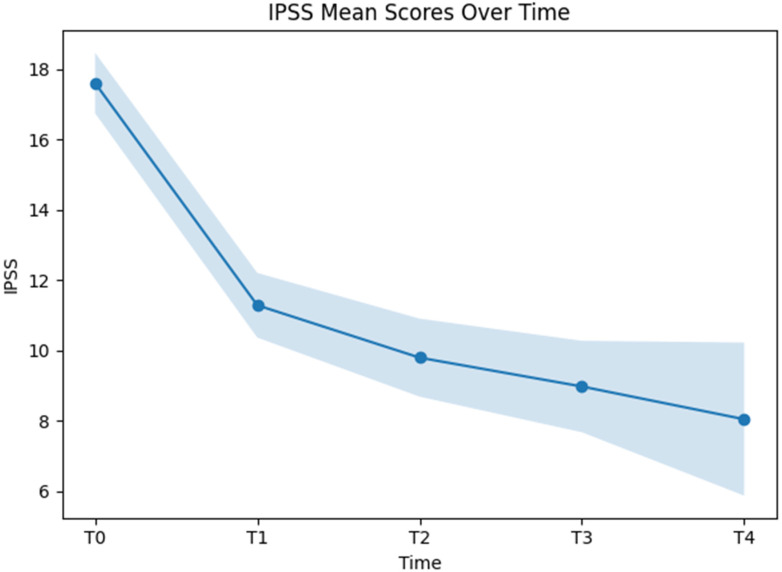
Linear mixed-effects modeled IPSS scores by time with time as a fixed factor.

The linear mixed-effects model with time as a fixed factor showed statistically significant IPSS scores across measurement time points (F(4, 515.25) = 89.77, *p* < 0.001). Estimated marginal means of the IPSS decreased over time, from 17.60 (95% CI: 16.74–18.46) at T0 to 11.28 (95% CI: 10.36–12.20) at T1, 9.79 (95% CI: 8.68–10.90) at T2, 8.97 (95% CI: 7.67–10.27) at T3, and 8.04 (95% CI: 5.87–10.22) at T4 (Figure 4).

Pairwise comparisons of estimated marginal means were performed using Bonferroni-adjusted tests to assess differences in IPSS scores between individual time points.

Compared with the baseline, IPSS scores were lower at all subsequent follow-up assessments. The mean difference between the baseline and 12 weeks was −6.32 points (95% CI: −7.60 to −5.04; *p* < 0.001). Differences between baseline and 24 weeks (−7.81 points; 95% CI: −9.37 to −6.25; *p* < 0.001), baseline and 36 weeks (−8.62 points; 95% CI: −10.46 to −6.79; *p* < 0.001), and baseline and 48 weeks (−9.56 points; 95% CI: −12.66 to −6.46; *p* < 0.001) were also statistically significant.

When comparing follow-up time points, the difference between T1 and T2 was −1.49 points (95% CI: −3.09 to 0.11; *p* = 0.090) and did not reach statistical significance. In contrast, IPSS scores at T3 were lower than at T1, with a mean difference of −2.31 points (95% CI: −4.18 to −0.43; *p* = 0.006). Also, the comparison between T1 and T4 showed a statistically significant difference (−3.24 points; 95% CI: −6.37 to −0.11; *p* = 0.037).

No statistically significant differences were observed between T2 and T3 (−0.82 points; 95% CI: −2.83 to 1.19; *p* = 1.000), between T2 and T4 (−1.75 points; 95% CI: −4.96 to 1.46; *p* = 1.000), or between T3 and T4 (−0.93 points; 95% CI: −4.25 to 2.38; *p* = 1.000).

The random-effects component indicated between-patient variability, with an estimated random intercept variance of 12.78 (SE 2.27), while the residual variance was estimated at 21.18 (SE 1.35). The intraclass correlation coefficient was 0.38 for the unconditional model and 0.27 for the conditional model. Marginal and conditional pseudo-R^2^ values were 0.28 and 0.55, respectively.

When study arm was included as an additional factor, the main effect of time on IPSS scores remained highly significant (F(4, 508.26) = 86.52, *p* < 0.001). A significant overall difference between study arms was observed (F(2, 568.26) = 7.20, *p* < 0.001), indicating lower mean IPSS values in the intervention group compared with the control group across the observation period. The interaction between study arm and time was not statistically significant (F(6, 503.42) = 1.83, *p* = 0.091), suggesting that the pattern of IPSS change over time did not differ substantially between groups. Estimated marginal means supported these findings, with consistently lower IPSS scores in the intervention group, particularly at earlier time points, while both groups showed similar temporal trajectories of symptom improvement. Substantial between-patient variability persisted, as reflected by the variance components (random intercept variance: 12.90; residual variance: 20.63). The marginal and conditional pseudo-R^2^ values were 0.30 and 0.57, respectively.

When adherence was instead included as an additional fixed factor alongside time, and the interaction between time and adherence was modeled, the linear mixed-effects analysis showed no statistically significant main effect of time on IPSS scores (F(4, 240.80) = 0.85, *p* = 0.492). Also, neither the main effect of adherence (F(5, 244.79) = 1.67, *p* = 0.142) nor the time-by-adherence interaction (F(14, 241.54) = 1.37, *p* = 0.168) reached statistical significance. Pairwise comparisons of estimated marginal means, adjusted for multiple testing, did not reveal statistically significant differences between adherence levels at individual time points or across time within adherence strata. The conditional pseudo-R^2^ of the model was 0.65, while the marginal pseudo-R^2^ was 0.06, indicating that a substantial proportion of variance was attributable to between-patient differences captured by the random intercept. The intraclass correlation coefficient was 0.62 in the adjusted model.

#### 3.2.2. Responder Analysis

Responder rates were assessed at each follow-up time point using two predefined thresholds for minimal important difference (MID), namely an IPSS reduction of ≥3 points and ≥5.26 points.

For the MID threshold of 3 points, 66.7% of patients were classified as responders at T1. The proportion of responders increased to 84.3% at T2 and 87.3% at T3 and was 80.1% at T4. Across all time points combined, 79.4% of patients met the responder criterion comparing individual last observations with T0, while 20.6% of those patients did not.

Using the more stringent MID threshold of 5.26 points, 46.6% of patients were classified as responders at T1. Responder proportions increased to 62.0% at T2, 73.2% at T3, and 76.2% at T4.

Responder rates by time point and MID threshold are summarized in Table 3.

#### 3.2.3. Persistence of Effects—IPSS After Discontinuation of App-Based Therapy

The course of IPSS scores following discontinuation of app-based therapy was analyzed using a linear mixed-effects model with the time since therapy discontinuation included as a categorical fixed effect with three levels. Patient-specific random intercepts were specified to account for within-subject correlation. In total, there were n = 36 patients at 3 months, 12 patients at 6 months, and 3 patients at 9 months after discontinuation that completed the examinations.

**Figure 5:**
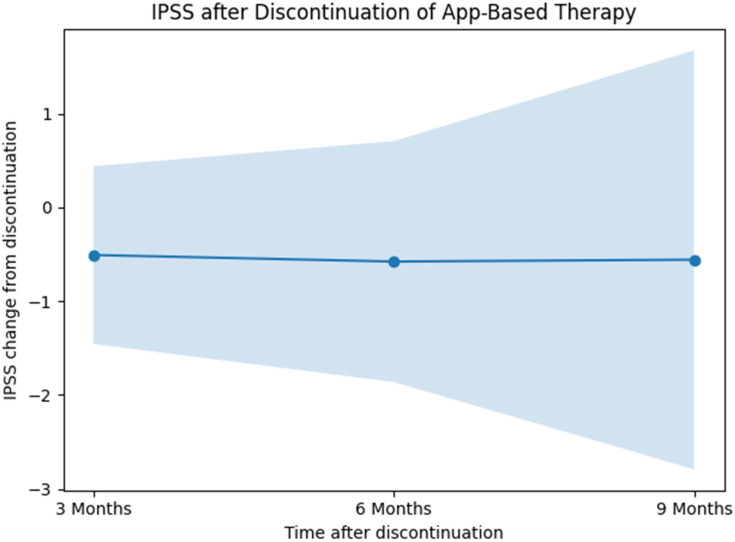
Linear mixed-effects modeled change of IPSS score from discontinuation of app use by time with time as a fixed factor.

The fixed effect of time since discontinuation was not statistically significant (F(2, 19.75) = 0.01, *p* = 0.99), indicating no differences in IPSS scores across the post-discontinuation time points. The estimated marginal mean IPSS change after therapy discontinuation was −0.55 (95% CI: −1.69 to 0.59). Estimated marginal means for the individual post-discontinuation time points were −0.51 (95% CI: −1.46 to 0.44) at the first assessment, −0.58 (95% CI: −1.86 to 0.71) at the second assessment, and −0.56 (95% CI: −2.79 to 1.68) at the third assessment (Figure 5). Bonferroni-adjusted pairwise comparisons did not reveal statistically significant differences between any of the post-discontinuation time points.

The random-effects component indicated substantial between-patient variability, with an estimated random intercept variance of 4.73 (SE 1.78) and a residual variance of 2.22 (SE 0.77). The intraclass correlation coefficient was 0.68 for both the unconditional and conditional models. Marginal pseudo-R^2^ was 0.00, while conditional pseudo-R^2^ was 0.68.

### 3.3. Secondary Efficacy Variables

#### 3.3.1. OAB-q-SF

Using the same linear mixed-effects modeling approach as for IPSS, the longitudinal course of OAB-q SF1 and OAB-q-SF2 scores were analyzed, with time included as a categorical fixed factor (T0–T4) and patient-specific random intercepts.

#### 3.3.2. OAB-q-SF1

**Figure 6:**
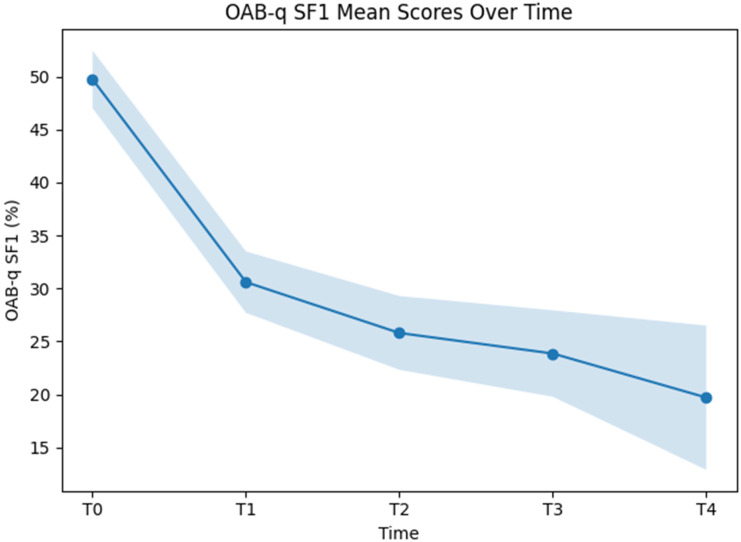
Linear mixed-effects modeled OAB-q-SF 1 scores by time with time as a fixed factor.

The fixed effect of time reached statistical significance (F(4, 524.60) = 85.37, *p* < 0.001), demonstrating variation in OAB-q SF1 scores across the assessed time points. Estimated marginal means showed a progressive decline over time, with values of 49.74 (95% CI: 47.01–52.46) at T0, 30.59 (95% CI: 27.69–33.49) at T1, 25.79 (95% CI: 22.31–29.28) at T2, 23.85 (95% CI: 19.77–27.93) at T3, and 19.70 (95% CI: 12.90–26.49) at T4 (Figure 6).

Relative to baseline, OAB-q SF1 scores were significantly lower at all subsequent follow-up assessments. The mean difference between T0 and T1 was −19.14 percentage points (95% CI: −23.13 to −15.16; *p* < 0.001). Corresponding differences were −23.94 points at T2 (95% CI: −28.80 to −19.08; *p* < 0.001), −25.89 points at T3 (95% CI: −31.62 to −20.15; *p* < 0.001), and −30.04 points at T4 (95% CI: −39.73 to −20.36; *p* < 0.001).

Comparisons between follow-up assessments showed that the difference between T1 and T2 was −4.80 points (95% CI: −9.79 to 0.19; *p* = 0.069) and did not reach statistical significance. In contrast, scores at T3 were lower than at T1, with a mean difference of −6.74 points (95% CI: −12.58 to −0.91; *p* = 0.012). A statistically significant difference was also observed between T1 and T4 (−10.90 points; 95% CI: −20.65 to −1.14; *p* = 0.017).

No statistically significant differences were detected between T2 and T3 (−1.94 points; 95% CI: −8.21 to 4.32; *p* = 1.000), between T2 and T4 (−6.10 points; 95% CI: −16.11 to 3.91; *p* = 0.866), or between T3 and T4 (−4.16 points; 95% CI: −14.50 to 6.19; *p* = 1.000).

The random-effects structure reflected between-patient variability, with an estimated variance of the random intercept of 129.84 (SE 22.25) and a residual variance of 205.69 (SE 12.97). The intraclass correlation coefficient was 0.39 for the unconditional model and 0.28 for the conditional model. Marginal and conditional pseudo-R^2^ values were 0.27 and 0.55, respectively.

#### 3.3.3. OAB-q-SF2

**Figure 7:**
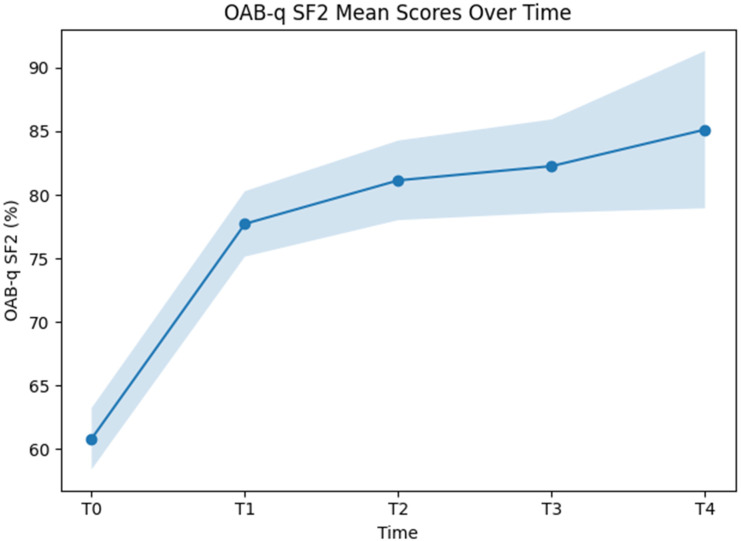
Linear mixed-effects modeled OAB-q-SF 2 scores by time with time as a fixed factor.

Applying a linear mixed-effects model to OAB-q SF2 outcomes with time as a fixed effect revealed statistical significance (F(4, 513.02) = 73.34, *p* < 0.001), indicating differences in scores across assessment time points. Estimated marginal means increased over time, with values of 60.83 (95% CI: 58.42–63.24) at T0, 77.72 (95% CI: 75.14–80.29) at T1, 81.14 (95% CI: 78.01–84.27) at T2, 82.26 (95% CI: 78.59–85.93) at T3, and 85.13 (95% CI: 78.94–91.32) at T4 (Figure 7).

Relative to the baseline, OAB-q SF2 scores were higher at all follow-up assessments. The mean difference between T0 and T1 was 16.89 percentage points (95% CI: 13.23 to 20.55; *p* < 0.001). Differences between the baseline and T2 (20.31 points; 95% CI: 15.84 to 24.77; *p* < 0.001), baseline and T3 (21.43 points; 95% CI: 16.18 to 26.68; *p* < 0.001), and baseline and T4 (24.30 points; 95% CI: 15.43 to 33.17; *p* < 0.001) were also statistically significant.

Comparisons between follow-up assessments did not reveal statistically significant differences between adjacent or later time points. Specifically, no significant differences were observed between T1 and T2, T1 and T3, T1 and T4, or between later follow-up assessments.

The random-effects component reflected between-patient variability, with an estimated random intercept variance of 94.59 (SE 17.37) and a residual variance of 173.04 (SE 11.06). The intraclass correlation coefficient was 0.35 for the unconditional model and 0.27 for the conditional model. Marginal and conditional pseudo-R^2^ values were 0.25 and 0.52, respectively.

## 4. Discussion

To the best of our knowledge, this study is the first to evaluate differential short- and long-term effects of a multimodal digital intervention for the treatment of male lower urinary tract symptoms.

The primary finding of this study was a robust and sustained reduction in LUTS severity, as measured by the IPSS, across all follow-up time points up to 48 weeks. The largest symptom improvement occurred during the first 12 weeks of therapy, corresponding to the initial treatment cycle, with a mean IPSS reduction exceeding 6 points. This magnitude clearly surpassed the established minimal important difference thresholds and confirmed the clinical relevance of the intervention. Importantly, further symptom reductions were observed with additional therapy cycles, although the incremental gains diminished over time, suggesting a pattern of early response followed by gradual stabilization.

This temporal pattern was mirrored in both OAB-q SF symptom severity and quality-of-life scores. For both secondary endpoints, substantial improvements were observed early, with continued but smaller numerical improvements during later follow-up. The absence of statistically significant differences between adjacent later time points supports the interpretation that symptom control is largely achieved within the first treatment cycle, while subsequent cycles may consolidate and maintain these effects rather than induce pronounced additional improvement.

When study arm was included in the mixed-effects models, lower overall IPSS scores were observed in the intervention group compared with the control group across the observation period. However, the absence of a statistically significant time-by-study-arm interaction indicates that both groups followed comparable trajectories once treatment was initiated. The similarity of symptom trajectories across study arms suggests that the observed improvements are attributable to the intervention itself rather than contextual effects related to early versus delayed access. At the same time, the persistently lower symptom levels in the initially treated group may reflect subtle differences in early care intensity, patient expectations, or unmeasured behavioral factors.

Adherence to the digital intervention showed a clear and progressive decline across therapy cycles, with frequent app use most common during the first cycle and non-use predominating by the fourth cycle. This pattern was observed in both study arms. Importantly, despite this decline in app usage, symptom improvements were sustained, and no significant association between adherence level and IPSS outcomes was detected by explorative use of mixed-effects models. The lack of statistically significant main or interaction effects involving adherence should be interpreted cautiously. Adherence was measured as a nominal categorical variable reflecting usage frequency rather than therapeutic intensity or fidelity, and the distance between categories cannot be assumed to be equidistant. Moreover, declining app use may not necessarily indicate disengagement from the therapeutic content but could reflect increasing internalization of behavioral strategies and reduced reliance on app-based guidance. This interpretation is slightly supported by the persistence of treatment effects after app discontinuation.

The absence of evidence for changes in IPSS scores up to 36 weeks after stopping app use could indicate that treatment effects were largely maintained over time. This finding suggests that patients may successfully transfer learned behavioral strategies into daily routines, resulting in sustained symptom control without ongoing digital support. Due to the small sample size underlying this analysis, these results should be interpreted cautiously. From a clinical perspective, these results are highly relevant, as they imply that recurrent continuous app engagement may not be necessary to preserve benefits once therapeutic routines are established. This has important implications for patient counselling, long-term adherence expectations, and the cost-effectiveness of digital interventions.

Responder analyses further contextualize the clinical relevance of the observed effects. Depending on the applied MID threshold, between two thirds and over three quarters of patients were classified as responders at later follow-up time points. The increasing responder rates over time, particularly when using the more stringent MID threshold, indicate that cumulative exposure to the intervention increases the likelihood of achieving clinically meaningful symptom improvement.

Notably, responder proportions remained high at T4, despite reduced app usage and partial attrition, reinforcing the notion of durable benefit in a substantial proportion of patients.

Few studies have examined the long-term effects of self-management interventions associated with lower urinary tract symptoms. In a systematic review, Hall et al. [9] analyzed 108 training programs, reporting intervention durations ranging from 1 to 52 weeks; however, duration was not specified in 36 out of the included studies, similar to the reporting of session number and length. In addition, the content of the interventions was highly heterogeneous across programs [9].

The only trial evaluating follow-up time points comparable to those of the present study was the randomized controlled trial by Brown et al. [10], in which patients were assessed at 3, 6, and 12 months after initiation of the intervention. The intervention consisted of lifestyle modifications, including fluid management and the avoidance of caffeine and alcohol, as well as behavioral strategies such as bladder retraining, double voiding, and urethral milking. Educational components were delivered in face-to-face small group sessions, and pelvic floor muscle training was not included. Both the intervention and control groups were followed for up to 12 months, with between-group differences evaluated at each time point. In that study, the between-group difference in IPSS was 5.7 points at 3 months, 6.5 points at 6 months, and 5.1 points at 12 months. Although an additional improvement was observed at 6 months, this effect was not sustained at the 12-month assessment, in contrast to the pattern observed in the present study. Other studies included in the systematic review by Albarqouni et al. [11] applied different intervention durations or did not include sequential follow-up assessments comparable to those used here. Comparisons across these heterogeneous studies suggest a trend favoring interventions of approximately 6 months’ duration [12,13] over shorter programs of up to 12 weeks [14,15,16], thereby partially supporting the findings of the present analysis.

The BEST study was the first to demonstrate a significant and clinically relevant improvement of LUTS after 12 weeks of a multimodal digital intervention [2]. The long-term findings presented in the current analysis suggest that continuous behavioral lifestyle change is associated with stabilizing improvements of symptom severity and quality of life.

This study has several limitations that should be considered when interpreting the findings. While the extended follow-up addresses a key limitation of the original study, namely its restriction to short-term outcomes [2], the absence of objective urological assessments, such as uroflowmetry or post-void residual volume measurements, remains. Nevertheless, evidence suggests that disease progression over periods exceeding four years is more frequently characterized by the worsening of symptoms (78% of patients) than by the development of incontinence or acute urinary retention (12% and 9% of patients, respectively) [17]. In addition, the observational nature of long-term follow-up and patient-driven continuation of therapy cycles introduce the potential for selection bias, as patients who remained engaged may differ systematically from those who discontinued early. Adherence measurement represents another methodological limitation. It was assessed using a categorical self-report measure with uneven intervals between response categories, which does not objectively measure training intensity, nor does it capture qualitative aspects of engagement, such as the correct execution of pelvic floor exercises or adherence to specific behavioral components of the multimodal program. Moreover, the adherence measure does not allow for differentiation between app-guided training and the autonomous continuation of exercises after discontinuation of active app use. This is particularly relevant considering the mixed-model analyses, suggesting a stabilization of symptoms over time and the absence of a strong independent adherence effect. It is therefore plausible that some patients internalized the training content and continued to benefit from the intervention without ongoing app interaction, leading to a potential underestimation of true long-term adherence and treatment exposure.

Taken together, these limitations highlight the need for future studies combining digital interventions with objective functional assessments, more granular adherence metrics, and predefined long-term follow-up strategies. Such designs would allow for a more precise disentanglement of treatment intensity, sustained behavioral change, and long-term symptom trajectories in digital multimodal therapy for male LUTS.

Given the multifactorial and heterogeneous nature of lower urinary tract symptoms, individualized and multimodal treatment strategies are increasingly recommended. The findings of the present study further support the integration of digital multimodal therapies into routine care pathways, either as a first-line treatment or in combination with established therapeutic modalities.

## 5. Conclusions

Given the multifactorial and heterogeneous nature of lower urinary tract symptoms, individualized and multimodal treatment strategies are increasingly recommended. This study demonstrates that a multimodal digital intervention for male LUTS induces a rapid and clinically meaningful symptom reduction within the first 12 weeks, with consolidating and sustaining benefits over up to 48 weeks, largely independent of continued app use, indicating durable behavioral treatment effects with high responder rates. The findings of the present study thereby support the integration of digital multimodal therapies into routine care pathways, either as a first-line treatment or in combination with established therapeutic modalities.

## Figures and Tables

**Table 1:** Baseline demographic and clinical characteristics of patients contributing data at each follow-up time point.

Parameter	Baseline (n = 237)	T1 (n = 189)	T2 (n = 108)	T3 (n = 71)	T4 (n = 21)
Mean Age (SD)	58.4 (±12.3)	58.6 (±11.8)	60.1 (±9.8)	60.6 (±8.6)	58.5 (±7.3)
Age ≤ 65 (%)	166 (70.0)	132 (69.8)	74 (68.5)	48 (67.6)	17 (81.0)
Age > 65 (%)	71 (30.0)	57 (30.2)	34 (31.5)	23 (32.4)	4 (19.0)
BMI	26.6 (±4.1)	26.4 (±3.9)	26.5 (±3.5)	26.4 (±3.1)	27.0 (±2.8)
**Disease Characteristics**					
Prostatic Hyperplasia—no. (%)	110 (46.4)	93 (49.2)	60 (55.56)	40 (56.3)	12 (57.1)
Other specified diseases of the urinary bladder—no. (%)	74 (31.2)	52 (27.5)	22 (20.4)	15 (21.1)	3 (14.3)
Combination prostatic hyperplasia (ICD code N40) and other specified diseases of the urinary bladder—no. (%)	53 (22.4)	44 (23.3)	26 (24.1)	16 (22.5)	6 (28.6)
Current medication for bladder voiding dysfunction—no. (%)	69 (29.1)	57 (30.2)	36 (33.3)	22 (31.0)	4 (19.0)
Previous therapies for bladder voiding dysfunction—no. (%)	50 (21.1)	42 (22.2)	28 (25.9)	19 (26.8)	6 (28.6)
IPSS Average (SD)	17.57 (±6.3)	17.63 (±6.2)	18.1 (±6.1)	18.14 (±6.2)	19.14 (±6.3)

BMI denotes body mass index (the weight in kilograms divided by the square of the height in meters); ICD, International Classification of Diseases.

**Table 2:** Longitudinal adherence patterns by treatment cycle in DIG and PIG.

	No Usage		With Longer Pauses Than 6 Weeks		With Longer Pauses Between 2–6 Weeks		With Longer Pauses Than 1 Week		Weekly		Multiple Times a Week	
	DIG	PIG	DIG	PIG	DIG	PIG	DIG	PIG	DIG	PIG	DIG	PIG
TC1	0.00%	6.15%	2.83%	6.15%	2.83%	7.69%	4.72%	10.77%	5.66%	0.00%	83.96%	69.23%
TC2	19.30%	12.50%	10.53%	12.50%	8.77%	15.00%	7.02%	10.00%	5.26%	7.50%	49.12%	42.50%
TC3	44.74%	42.86%	15.79%	17.86%	5.28%	10.71%	0.00%	0.00%	10.53%	10.71%	23.68%	17.86%
TC4	61.90%		4.76%		4.76%		4.76%		9.52%		14.29%	

**Table 3:** Proportion of IPSS responders over time according to predefined minimal important difference (MID) thresholds.

MID	Responder	T1	T2	T3	T4
3	Yes (%)	126 (66.7)	91 (84.3)	62 (87.3)	17 (80.1)
	No (%)	63 (33.3)	17 (15.7)	9 (12.7)	4 (19.9)
5.26	Yes (%)	88 (46.6)	67 (62.0)	52 (73.2)	16 (76.2)
	No (%)	101 (53.4)	41 (38.0)	19 (26.8)	5 (23.8)

## Data Availability

The raw data supporting the conclusions of this article may be made available by the authors on request.
